# Hepcidin Decreases Rotenone-Induced α-Synuclein Accumulation *via* Autophagy in SH-SY5Y Cells

**DOI:** 10.3389/fnmol.2020.560891

**Published:** 2020-10-16

**Authors:** Meiqi Li, Jianan Hu, Xiaoyu Yuan, Lihua Shen, Li Zhu, Qianqian Luo

**Affiliations:** ^1^Department of Physiology and Hypoxic Biomedicine, Institute of Special Environmental Medicine and Co-Innovation Center of Neuroregeneration, Nantong University, Nantong, China; ^2^Department of Emergency, Affiliated Hospital of Nantong University, Nantong, China; ^3^Department of Neurology, Affiliated Hospital of Nantong University, Nantong, China

**Keywords:** hepcidin, α-synuclein, iron, rotenone, Parkinson’s disease (PD)

## Abstract

Parkinson’s disease (PD) is a neurodegenerative disorder, and the hallmarks of this disease include iron deposition and α-synuclein (α-syn) aggregation. Hepcidin could reduce iron in the central and peripheral nervous systems. Here, we hypothesized that hepcidin could further decrease α-syn accumulation *via* reducing iron. Therefore, rotenone or α-syn was introduced into human neuroblastoma SH-SY5Y cells to imitate the pathological progress of PD *in vitro*. This study investigated the clearance effects of hepcidin on α-syn induced by a relatively low concentration of rotenone exposure or α-syn overexpression to elucidate the potential clearance pathway involved in this process. We demonstrated that SH-SY5Y cell viability was impaired after rotenone treatment in a dose-dependent manner. α-syn expression and iron content increased under a low concentration rotenone (25 nM for 3 days) treatment in SH-SY5Y cells. Pre-treatment with hepcidin peptide suppressed the abovementioned effects of rotenone. However, hepcidin did not affect treatment with rotenone under high iron conditions. Hepcidin also played a role in reducing α-syn accumulation in rotenone and α-syn overexpression conditions. We identified that the probable clearance effect of hepcidin on α-syn was mediated by the autophagy pathway using pretreatment with autophagy inhibitors (3-MA and CQ) and detection of autophagy protein markers (LC3II/I and p62). In conclusion, hepcidin eliminated α-syn expression *via* the autophagy pathway in rotenone-treated and α-syn overexpression SH-SY5Y cells. This study highlights that hepcidin may offer a potential therapeutic perspective in α-syn accumulation diseases.

## Introduction

Parkinson’s disease (PD) is a progressive nervous disorder, and it is the most common movement disorder. The characteristics of PD include the selective loss of dopaminergic neurons in the substantia nigra pars compacta (SNpc; Dauer and Przedborski, 2003). The histological hallmark of PD is Lewy bodies (LB), of which the main constituent is fibrillar α-synuclein (α-syn; Spillantini et al., 1997). The pathogenesis of PD is poorly understood, but combinations of environmental and genetic factors are likely related to the etiology of PD (von Bohlen und Halbach et al., 2004). Rotenone has been used as a natural pesticide and insecticide worldwide for a long time. Rotenone is a naturally occurring compound derived from the roots of the Derris and Lonchocarpus plant species (Jenner, 2001), and it is an inhibitor of mitochondrial complex I. Rotenone induces the formation of LB-like inclusion bodies, which results in the selective degeneration of nigral dopamine neurons (Betarbet et al., 2000). α-syn protein is the main constituent of LB, and it is abundant in neurons as a 140-amino-acid protein. α-syn is primarily located in the presynaptic terminals. The accumulation and aggregation of α-syn in susceptible neurons is the trigger factor of dopamine neuron degeneration in PD (Dawson and Dawson, 2003; Sung et al., 2007). Iron may bind with α-syn (Bharathi et al., 2007; Peng et al., 2010), accelerate α-syn aggregation into fibrils and eventually contribute to the formation of LB (Paik et al., 2000; Golts et al., 2002). Several studies confirmed selective and significant elevations in iron content in the SNpc of patients with PD (Sofic et al., 1988; Kienzl et al., 1999).

Hepcidin is a liver-secreted hormone that plays a critical role in iron homeostasis in the peripheral and central nervous systems (Park et al., 2001; Nemeth and Ganz, 2006; Zechel et al., 2006). Our previous study demonstrated that hepcidin reduced brain iron in iron-overloaded rats *via* the down-regulation of iron transport protein Fpn1 (Du et al., 2015; Gong et al., 2016).

Therefore, we hypothesized that hepcidin could inhibit α-syn accumulation by reducing iron content. The present study used human neuroblastoma SH-SY5Y cells to introduce α-syn (genetic factor of PD) and rotenone (environmental factor of PD). We studied the role of hepcidin in the expression and accumulation of α-syn in the cell model and examined the mechanisms involved in this process. We demonstrated that 3-day exposure to relatively low concentrations of rotenone (25 nM) induced α-syn expression and an increase in iron content significantly in SH-SY5Y cells. Hepcidin removed rotenone-induced α-syn expression and accumulation probably *via* the autophagy pathway in rotenone-induced and α-syn overexpression conditions.

## Materials and Methods

### Materials

Unless otherwise related, all chemicals in this study including carbobenzoxy-Leu-Leu-leucinal (MG132), Chloroquine (CQ), 3-Methyladenine (3MA) were supplied by the Sigma Chemical Company (St. Louis, MO, USA). Synthetic human hepcidin peptide (25 amino acids) was purchased from Peptides International (Louisville, KY, USA). Anti-α-syn and mouse monoclonal anti-actin was obtained from Abcam (Cambridge, MA, USA). goat anti-mouse or anti-rabbit IRDye CW secondary antibodies from LI-COR BioSciences (Lincoln, NE, USA). Alexa Fluor 555 IgG from Life Technologies (Carlsbad, CA, USA); BCA Protein Assay Kits from Thermo Fisher Scientific (Pierce, MA, USA). RIPA lysis buffer from the Beyotime Institute of Biotechnology (Haimen, Jiangsu, China).

### Cell Viability Evaluation

SH-SY5Y cells were plated at a density of 3 × 10^3^ cells per well in 96-well plates for 12 h. For cell viability assay, SH-SY5Y cells were cultured in rotenone-containing media at a final concentration from 0 to 500 nM for 3 days. Following exposure to rotenone, cell viability was examined using an improved MTT assay, as we described previously (Luo et al., 2016). In brief, MTT (1 g/l in PBS, 25 μl) was added to each well before a 37°C incubation for 4 h. The assay was blocked with the addition of pH 4.7 lysis buffer (20% SDS in 50% N′N-dimethylformamide, 100 μl). Optical densities (OD) at 570 nm and 630 nm wavelengths were detected using a Synergy2 microplate assay reader (Bio-Tek, Winooski, VT, USA), and the results are expressed as a percentage of the absorbance measured in the control cells.

### Western Blot

Western blot was performed as described in our previous study (Luo et al., 2011). Protein contents were examined by the Pierce BCA protein assay kit. Thirty microgram protein of each group sample was loaded and ran in 10% SDS–PAGE gel under reducing conditions. The gel was subsequently transferred to the 0.45 μm PVDF membrane (Millipore, Burlington, MA, USA) after running. The membrane was blocked in 5% non-fat milk followed by incubating with primary antibodies against anti-α-syn (1:500), anti-actin (1:1,000), anti-LC3I/II (1:1,000) and anti-p62 (1:1,000) under 4°C overnight. The blots were incubated with goat anti-rabbit or anti-mouse IRDye 800 CW secondary antibody (1:10,000) for 2 h at room temperature after being washed for three times. The intensity of the specific blots was detected and analyzed by the Odyssey infrared imaging system (LI-COR Biosciences, Lincoln, NE, USA).

### Graphite Furnace Atomic Absorption Spectrophotometer (GFAAS)

The total iron content in SH-SY5Y cells after treatment with FeSO_4_ or FAC in the presence of rotenone was detected using a graphite furnace atomic absorption spectrophotometer (GFAAS, Perkin Elmer, Analyst 100), as previously described in other studies (Chang et al., 2005; Ke et al., 2005; Gong et al., 2016).

### Transient Transfection

SH-SY5Y cells were cultured on coverslips for 24 h and 3 μg pcDNA3.1-α-syn-GFP (pcDNA-syn) per well were transfected into the cell using X-treme GENE HP DNA transfection reagents (Roche, Basel, Switzerland). The same amount of pcDNA3.1-GFP plasmids per well was used as control. Following transfection, SH-SY5Y cells were incubated for an additional 24 h before further rotenone or hepcidin peptide treatment.

### Immunofluorescence

Immunofluorescence staining was performed as described in our previous work (Luo et al., 2018). Cells were fixed in 4% paraformaldehyde for 30 min followed by three washes in 0.01 M PBS. Then, the cells were incubated with PBS containing 3% bovine serum albumin (BSA) for 2 h, followed by incubation with a primary antibody against α-syn (1:200) overnight at 4°C. The cells were washed with PBS three times and incubated with the secondary antibody Alexa Fluor 555 goat anti-rabbit IgG (1:400) for 2 h at room temperature. The fluorescence signals were collected by a confocal fluorescence microscope (Carl Zeiss; Heidenheim, Germany) finally.

### Statistical Analysis

Statistical analyses were proceeded with Graphpad Prism 7.0 software. All the data in this study were presented as mean ± SEM. The variations among different groups were all examined by one-way analysis of variance (ANOVA) and followed by Tukey tests for *post hoc* comparisons. A *p*-value of less than 0.05 was considered statistically significant.

## Results

### Rotenone Induced α-Syn Expression in SH-SY5Y Cells

**Figure 1:**
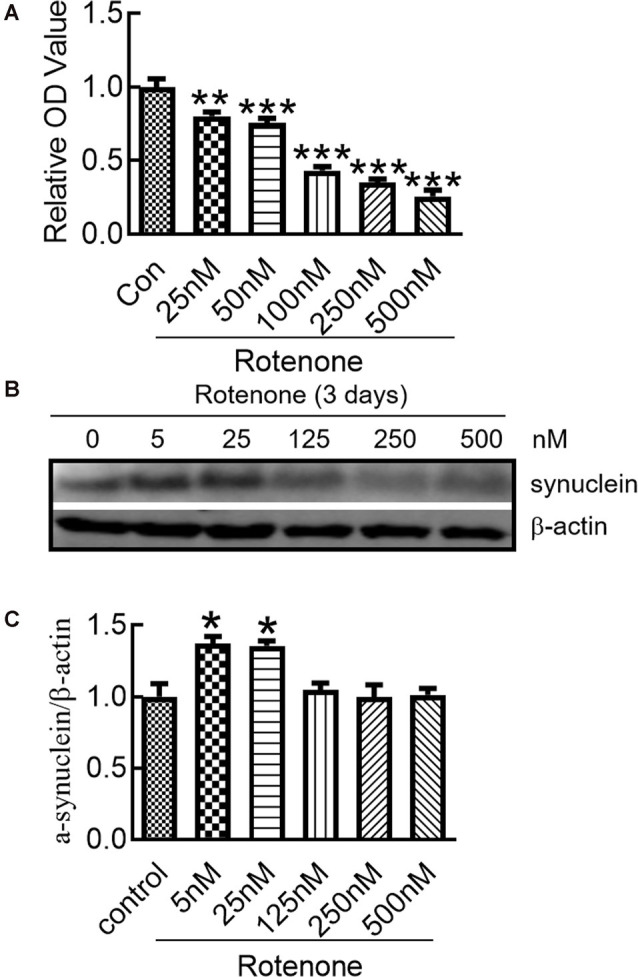
Changes in the α-syn expression in SH-SY5Y cells after rotenone treatment. **(A)** Cell viability of SH-SY5Y cells after treatment with different concentrations of rotenone for 3 days. **(B)** Western blot analysis of α-synuclein (α-syn) expression in SH-SY5Y cells after 0–500 nM rotenone treatment for 3 days. **(C)** Ratios of relative staining intensity for α-syn. *n* = 3 independent experiments. *p < 0.05 compared to the control. **p < 0.01; ***p < 0.001 compared to the control.

To evaluate the influence of rotenone on cell viability, the MTT assay was used to detect the viability of SH-SY5Y cells treated with different concentrations of rotenone for 3 days. The results demonstrated that rotenone significantly reduced cell viability in a dose-dependent manner (Figure 1A). We detected the effect of rotenone on the expression of α-syn in SH-SY5Y cells. The results showed that α-syn protein levels in the cells treated with 5 and 25 nM rotenone were markedly higher than those in the other groups (Figures 1B,C).

### Hepcidin Decreased α-Syn Expression and Iron Content in Rotenone-Treated SH-SY5Y Cells

**Figure 2:**
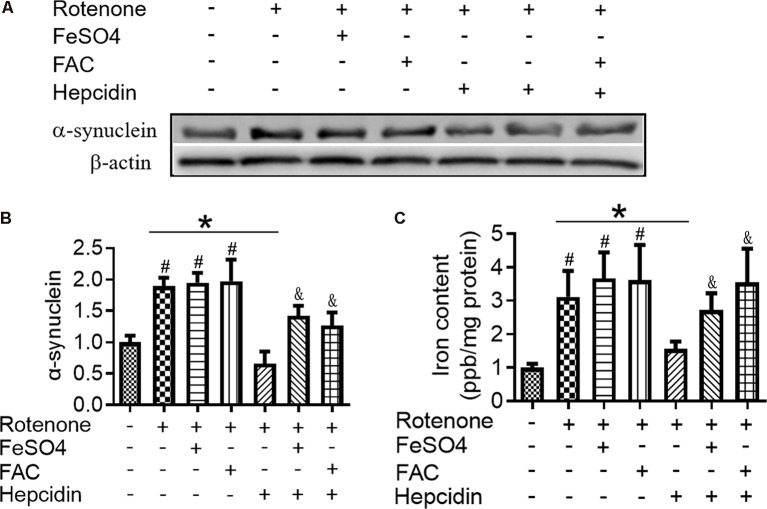
Hepcidin decreased α-syn expression in rotenone-induced and α-syn-overexpressed SH-SY5Y cells. SH-SY5Y cells were co-incubated with Ferric Ammonium Ciltete (FAC, 5 μM) or FeSO_4_ (5 μM) and rotenone (25 nM) for 3 days with or without hepcidin peptide (100 nM) co-incubation. **(A)** α-syn expression was determined using Western blotting. **(B)** Quantification of α-syn contents in panel **(A)**. **(C)** Total iron content in SH-SY5Y cells was determined using graphite furnace atomic absorption spectrophotometer (GFAAS) analysis as described in the “Materials and Methods” section. SH-SY5Y cells were transfected with 3 μg pcDNA-syn for 12 h followed by 100 nM hepcidin peptide treatment for 3 days. *n* = 3 independent experiments. *p < 0.05 compared to the indicated group. ^#^*p* < 0.05 compared to the control; ^&^*p* < 0.05 compared to the rotenone + hepcidin group.

**Figure 3:**
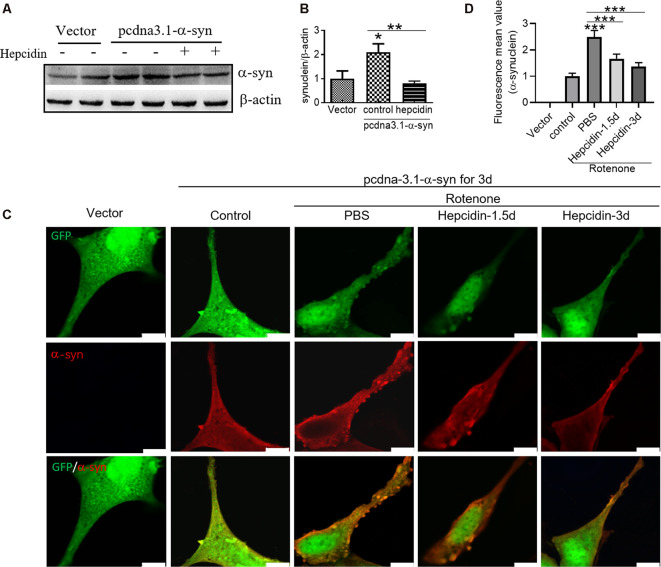
Hepcidin represses rotenone-induced α-syn expression and accumulation in α-syn-overexpressing SH-SY5Y cells. SH-SY5Y cells were transfected with 3 μg pcDNA-syn for 12 h and followed by 100 nM hepcidin peptide treatment for 1.5 or 3 days in the presence of 25 nM rotenone stimulation for 3 days. **(A)** α-syn expression was determined using Western blotting after hepcidin was treated for 3 days. **(B)** Quantification of α-syn contents in panel **(A)**. **(C)** Immunofluorescent detection showed the merge of α-synuclein-GFP (green) and labeling with α-syn antibodies (red), which revealed intracellular α-syn inclusions. **(D)** Quantification of the inclusions inflorescence value of the merged pictures. *n* = 3 independent experiments. *p < 0.05, ***p < 0.001 compared to the control; **p < 0.01, ***p < 0.001 compared to the indicated group. Bar = 100 μm.

To determine the effects of hepcidin on α-syn expression in SH-SY5Y cells, we investigated the role of hepcidin peptide on the expression of α-syn in physiological and iron-replete conditions. Figure 2 showed that iron and rotenone treatment induced an elevation of α-syn expression (Figure 2A) and iron content (Figure 2C). Hepcidin significantly decreased α-syn expression and iron content in the presence of rotenone. The expression of α-syn and iron content still increased significantly under iron-replete conditions (treatment with FAC or FeSO_4_) in the presence of hepcidin. However, hepcidin had no significant inhibition effect on α-syn or iron level in the presence of an overload of intracellular iron. To elucidate whether hepcidin exerted a similar influence under α-syn-overexpressing conditions, we observed the effect of hepcidin peptide on the expression of α-syn after transfecting SH-SY5Y cells with an α-syn recombination plasmid. After transfection with pcDNA-syn for 12 h and rotenone treatment for another 3 days, it showed a similar result that hepcidin peptide repressed the expression of α-syn significantly in the α-syn overexpression condition by western blot (Figures 3A,B). These results indicated that hepcidin peptide inhibited α-syn expression in rotenone-induced and α-syn-overexpression conditions.

### Hepcidin Reduced α-Syn Accumulation in Rotenone-Treated SH-SY5Y Cells

To verify whether rotenone-induced α-syn accumulation and to evaluate the effects of hepcidin on this process, we localized α-syn using fluorescence microscopy in α-syn-overexpressing SH-SY5Y cells. After transfection SH-SY5Y cells with pcDNA-α-syn plasmid for 12 h, rotenone was added to the cells for 3 days in the presence of 100 nM hepcidin peptide that had been incubated for 1.5 or 3 days. It was shown that the overexpression of α-syn in SH-SY5Y cells did not affect the accumulation of α-syn without rotenone treatment (Figures 3C,D). This result is consistent with a previous study (Ma et al., 2013). However, rotenone treatment for 3 days induced the formation of α-syn positive inclusions in most SH-SY5Y cells (Figures 3C,D, *p* < 0.001). The number of α-syn-containing inclusions in SH-SY5Y cells was reduced after treatment with hepcidin peptide for 1.5 or 3 days following 3 days of rotenone treatment, which suggests that hepcidin inhibits the accumulation of α-syn. These results indicate that rotenone promotes α-syn accumulation in SH-SY5Y cells, and hepcidin inhibited this effect.

### The Elimination Effect of Hepcidin in α-Syn Was Not Mediated by the Proteasome System but by the Autophagy Pathway

**Figure 4:**
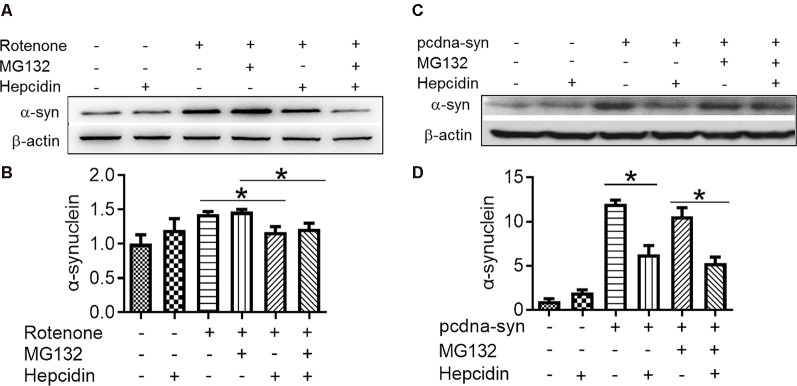
Hepcidin reduces rotenone-induced α-syn accumulation not *via* the proteasome system in rotenone-induced and α-syn-overexpressing SH-SY5Y cells. SH-SY5Y cells were transfected with or without 3 μg pcDNA-syn for 12 h, followed by 100 nM hepcidin peptide treatment with (+) or without (−) 1 h pre-incubation with MG132 (2.5 μM) in the presence of 25 nM rotenone stimulation for 3 days. **(A,C)** α-syn expression was determined using Western blotting. **(B,D)** Quantification of α-syn expression in panel **(A,C)**. *n* = 3 independent experiments. *p < 0.05 compared to the indicated group.

**Figure 5:**
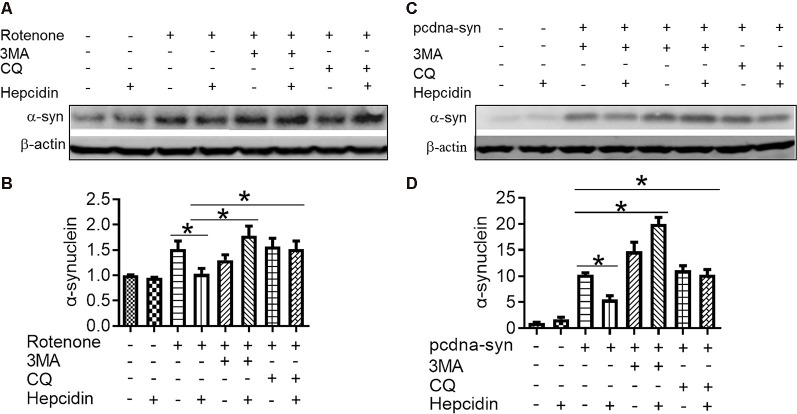
The elimination effect of hepcidin in rotenone-induced α-syn accumulation was mediated *via* the autophagy pathway in rotenone-induced and α-syn-overexpressing conditions. SH-SY5Y cells were transfected without or with 3 μg pcDNA-syn for 12 h, followed by 100 nM hepcidin peptide treatment with (+) or without (−) 1 h pre-incubation with 3MA (5 mM) or CQ (10 μM) in the presence of 25 nM rotenone stimulation for 3 days. **(A,C)** α-syn expression was detected using Western blotting. **(B,D)** Quantification of α-syn contents in panels **(A,C)**. *n* = 3 independent experiments. *p < 0.05 compared to the indicated group.

**Figure 6:**
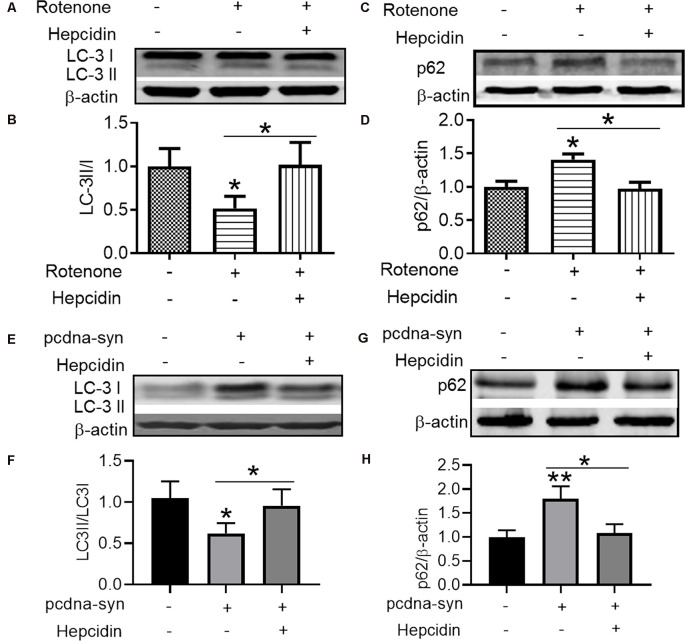
Hepcidin inhibits the decrease of autophagy-related protein expression in rotenone-induced and α-syn-overexpressed SH-SY5Y cells. **(A–D)** The SH-SY5Y cells were pre-treated with 100 nM hepcidin peptide followed by rotenone treatment for 3 days. **(E–H)** SH-SY5Y cells were transfected without or with 3 μg pcDNA-syn for 12 h, followed by 100 nM hepcidin peptide treatment. **(A,C,E,G)** LC3 and p62 expression were determined using Western blotting. **(B,D,F,H)** Quantification of the contents of LC3II/LC3I and p62 in panels **(A,C)**. *n* = 3 independent experiments. *p < 0.05, **p < 0.05 compared to the control; *p < 0.05 compared to the indicated group.

There are two crucial protein degradation systems in eukaryotic cells, the autophagy system, and the ubiquitin-proteasome system. To elucidate the clearance pathway involved in the effect of hepcidin on α-syn expression and accumulation, we detected the two systems by using specific inhibitors of the two systems in rotenone-induced and α-syn-overexpressed conditions (Figures 4–6). The proteasome inhibitor MG132 is a peptide aldehyde that effectively blocks the proteolytic activity of the proteasome complex (Lee and Goldberg, 1998). As shown in Figure 4, hepcidin reduced rotenone-induced α-syn accumulation in rotenone-induced and α-syn-overexpressed conditions. However, pre-incubation with MG132 for 1 h failed to inhibit the hepcidin-induced reduction of α-syn. The results indicated that the clearance effect of hepcidin on α-syn was not mediated *via* the proteasome pathway. We further tested whether the clearance effect of hepcidin on α-syn primarily occurred *via* the autophagy pathway by using autophagy inhibitors (CQ and 3MA). 3MA is a selective autophagy and phosphoinositide 3-kinase (PI3K) inhibitor (Wu et al., 2010), and chloroquine (CQ) increases the lysosomal pH, which further inhibits the fusion of the autophagosome with the lysosome (Shintani and Klionsky, 2004). As shown in Figure 5, hepcidin reduced α-syn accumulation in rotenone-induced and α-syn-overexpressed conditions. However, pre-incubation with 3MA or CQ for 1 h inhibited the hepcidin-induced reduction of α-syn. It was shown that rotenone inhibited the autophagy pathway in rotenone-induced and α-syn-overexpressed SH-SY5Y cells, as indicated by the lipid-mediated processing of the autophagy marker, the ratio of microtubule-associated protein 1A light chain protein 3B (LC3B-II) to LC3B-I (Figures 6A,B,E,F). These results suggested that hepcidin increased the LC3II/LC3I ratio significantly in both rotenone-induced and α-syn-overexpressed conditions. While the expression of p62 was reduced after incubation with hepcidin in both rotenone-induced and α-syn-overexpressed SH-SY5Y cells (Figures 6C,D,G,H). These results demonstrated that hepcidin stimulated the autophagy pathway in SH-SY5Y cells under the rotenone-induced or α-syn-overexpressed conditions. Taken together, these results indicated that the clearance effect of hepcidin on α-syn was primarily mediated *via* the autophagy pathway but not the proteasome system.

## Discussion

A large body of evidence suggests that α-syn aggregation occupies an important position in the pathogenesis of PD (Dawson and Dawson, 2002; Beal, 2003). α-Syn is also one of the molecular mechanisms involved in several other neurodegenerative disorders, including LB variant Alzheimer’s disease and dementia with LB (Burke, 2004; Davies et al., 2011). However, little is known about how the cells resolve these potentially toxic protein aggregates, such as α-syn.

To study the potential molecular mechanisms related to the degeneration of dopaminergic neurons in PD, rotenone was used to induce a PD model *in vitro* and *in vivo*. Although rotenone has frequently been used to investigate PD, the treatment concentration is different between studies (Lu et al., 2010; Yuan et al., 2015). At the beginning of this study, we first performed a dose course of rotenone to determine which concentration would induce α-syn expression significantly. We further investigated whether hepcidin affected α-syn expression. We demonstrated that continuous exposure of SH-SY5Y cells to different concentrations of rotenone for 3 days produced a dose-dependent decline in cell viability. However, the expression of α-syn was increased obviously after 5 or 25 nM rotenone incubation for 3 days. This result is similar to the results of other studies (Lu et al., 2010). Although most results in rotenone-induced dopaminergic cell cultures used short-term and relatively high concentrations of rotenone exposure, PD patients are likely exposed to the toxins for several years at relatively low concentrations. Therefore, the rotenone concentration used in the present study was more reasonable and close to clinical exposure. These results indicated that chronic low-grade rotenone exposure induced the expression of α-syn *in vitro*.

Previous investigations revealed a potentially significant association in the two characteristic molecular features found in PD, which are the accumulation of α-syn and the increased content of iron in the SNpc (Binolfi et al., 2006; Bharathi and Rao, 2007). There was also strong evidence that showed α-syn, as a cellular ferrireductase, was important for reducing iron (III) to bioavailable iron (II) (Davies et al., 2011). This knowledge prompted us to consider whether rotenone affected iron content and α-syn expression. Therefore, we postulated that hepcidin would diminish α-syn expression *via* a decrease in iron levels in rotenone-induced SH-SY5Y cells. We first investigated the effects of hepcidin on α-syn expression and total iron content in SH-SY5Y cells treated with rotenone in the presence or absence of iron. We confirmed for the first time that hepcidin significantly suppressed the rotenone-induced increase in α-syn expression and intracellular total iron content. These results strongly support our hypothesis that hepcidin had a significant effect in repressing SH-SY5Y cells from rotenone-induced α-syn expression and iron overload. However, our finding was conflicting with the results of a previous study (Xu et al., 2016), which showed that knockdown of hepcidin protected N27 cells from 6-OHDA-induced apoptosis by possibly regulating cellular iron export. Here, the expression of the iron exporter (Ferroportin, Fpn) was not changed significantly after treating with hepcidin alone or in the presence of rotenone in our study (data not shown). It was suggested that the inhibition effect of hepcidin on α-syn expression and cellular iron content in the presence of rotenone may not be mediated by Fpn. As expected, more in-depth mechanistic experiments and analyses showing how iron metabolism and autophagy should be involved in this process.

The accumulation of α-syn is a hallmark feature of PD, and it is referred to in other synucleinopathies. Metals are significant aetiological factors in PD. The status of mental interaction with α-syn affects the kinetics of fibrillation dramatically *in vitro*, and this interaction may exert important and potentially neurodegenerative effects *in vivo* (Bharathi and Rao, 2007; Bharathi et al., 2007; Peng et al., 2010). A previous study suggested that iron acted in concert with α-syn to prompt the formation of LB and induce cell death in PD (Ostrerova-Golts et al., 2000). The reduction or removal of α-syn aggregates may be a therapeutic strategy in the clinic. Evidence supports the existence of proteasome subunits in LB (Ii et al., 1997), which led us to propose that an impairment of the ubiquitin-proteasome system was relevant to the progression of PD. Some studies suggested that the inhibition of proteasomal caused a significant accumulation of α-syn, inclusion formation, and increased cell death (Bennett et al., 1999; Tofaris et al., 2001; McNaught et al., 2002), but other studies demonstrated that α-syn was not a proteasome substrate (Ancolio et al., 2000; Rideout et al., 2001). The autophagy pathway is also involved in α-syn clearance (Lee et al., 2004), and it mediates the bulk degradation of organelles in the lytic compartment or cytoplasmic proteins (Klionsky and Ohsumi, 1999; Ravikumar et al., 2002).

To elucidate whether the proteasome system and autophagy pathway were relevant to the hepcidin clearance effect, we examined the role of hepcidin in the expression of α-syn in the presence or absence of a proteasome system inhibitor (MG132) or autophagy pathway inhibitor (3MA and CQ) in rotenone-induced SH-SY5Y cells. We found that hepcidin decreased α-syn content significantly in rotenone-induced and α-syn overexpression conditions. However, the positive clearance effects of hepcidin on the expression of α-syn in rotenone-induced SH-SY5Y cells was not inhibited in the presence of the proteasome inhibitor MG132, but the positive repression effect of hepcidin on α-syn was inhibited after treatment with the autophagy inhibitors 3MA and CQ. Western blot analysis of LC3 and p62 also confirmed that rotenone and α-syn-overexpressed treatment inhibited the autophagy pathway, and pretreatment with hepcidin peptide increased the capacity of autophagy in SH-SY5Y cells. Some studies showed that both the proteasome system and the autophagy pathway eliminated α-syn (Webb et al., 2003). However, our findings support the hypothesis that hepcidin clears α-syn accumulation only *via* the autophagy pathway. The results further fundamentally help us understand the function of hepcidin on α-syn accumulation pathophysiology. However, the precise molecular physiological and pathological roles of hepcidin on the elimination of α-syn must be determined.

Taken together, our study demonstrated that rotenone-induced the expression and accumulation of α-syn and increased iron content in rotenone-induced and α-syn-overexpression conditions. Hepcidin not only decreased the expression and accumulation of α-syn induced by rotenone but also prompted the clearance of α-syn *via* the autophagy pathway. This elimination capacity of hepcidin offers a therapeutic perspective to not only prevent but also reverse the disease progression in PD and other disorders associated with α-syn high expression or accumulation.

## Data Availability Statement

All datasets presented in this study are included in the article/Supplementary Material.

## Author Contributions

QL and LZ conceived, organized, and supervised the work. ML, XY, and JH performed the experiments. JH and LS contributed to the analysis of data. QL prepared, wrote and revised the manuscript. All authors contributed to the article and approved the submitted version.

## Conflict of Interest

The authors declare that the research was conducted in the absence of any commercial or financial relationships that could be construed as a potential conflict of interest.
