# Supplementary material for: Hepcidin Decreases Rotenone-Induced α-Synuclein Accumulation via Autophagy in SH-SY5Y Cells
Source: Front Mol Neurosci. 2020 Oct 16;13:560891. doi: 10.3389/fnmol.2020.560891 (PMC7596286; doi:10.3389/fnmol.2020.560891)
Supplement: Supplementary file 2 [file Data_Sheet_2.ZIP › Supplementary data/Supplementary Figures.docx]

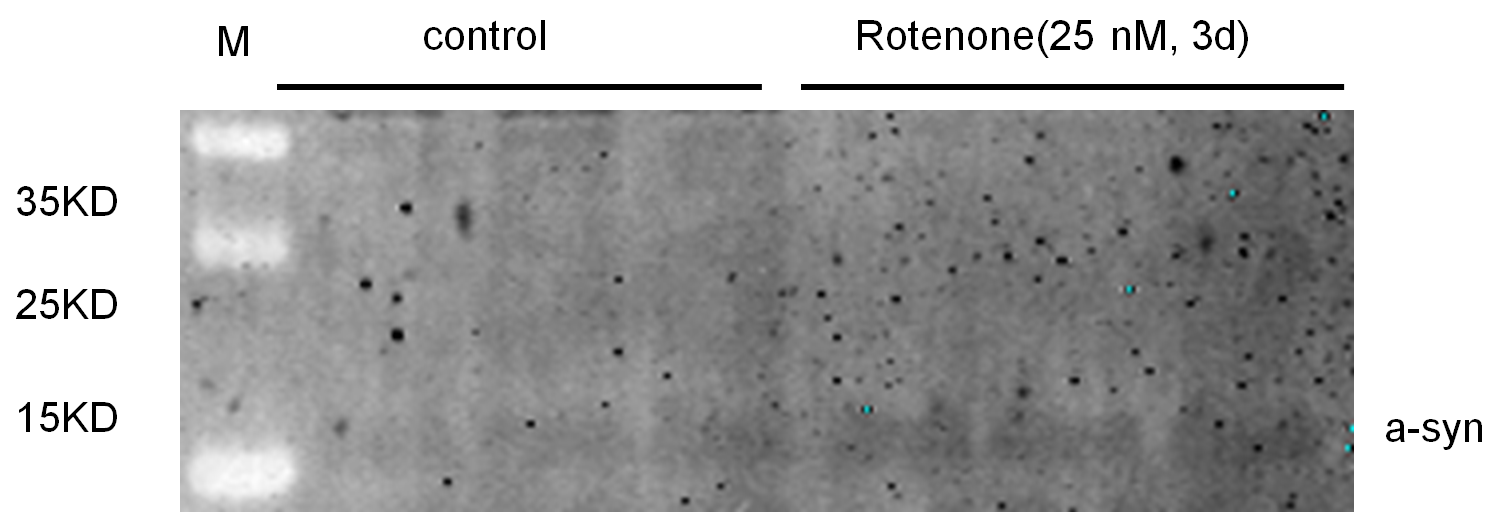


**Fig. S1** Western blot analysis of α-syn expression in MES23.5 cells after 25 nM rotenone treatment for 3 days.


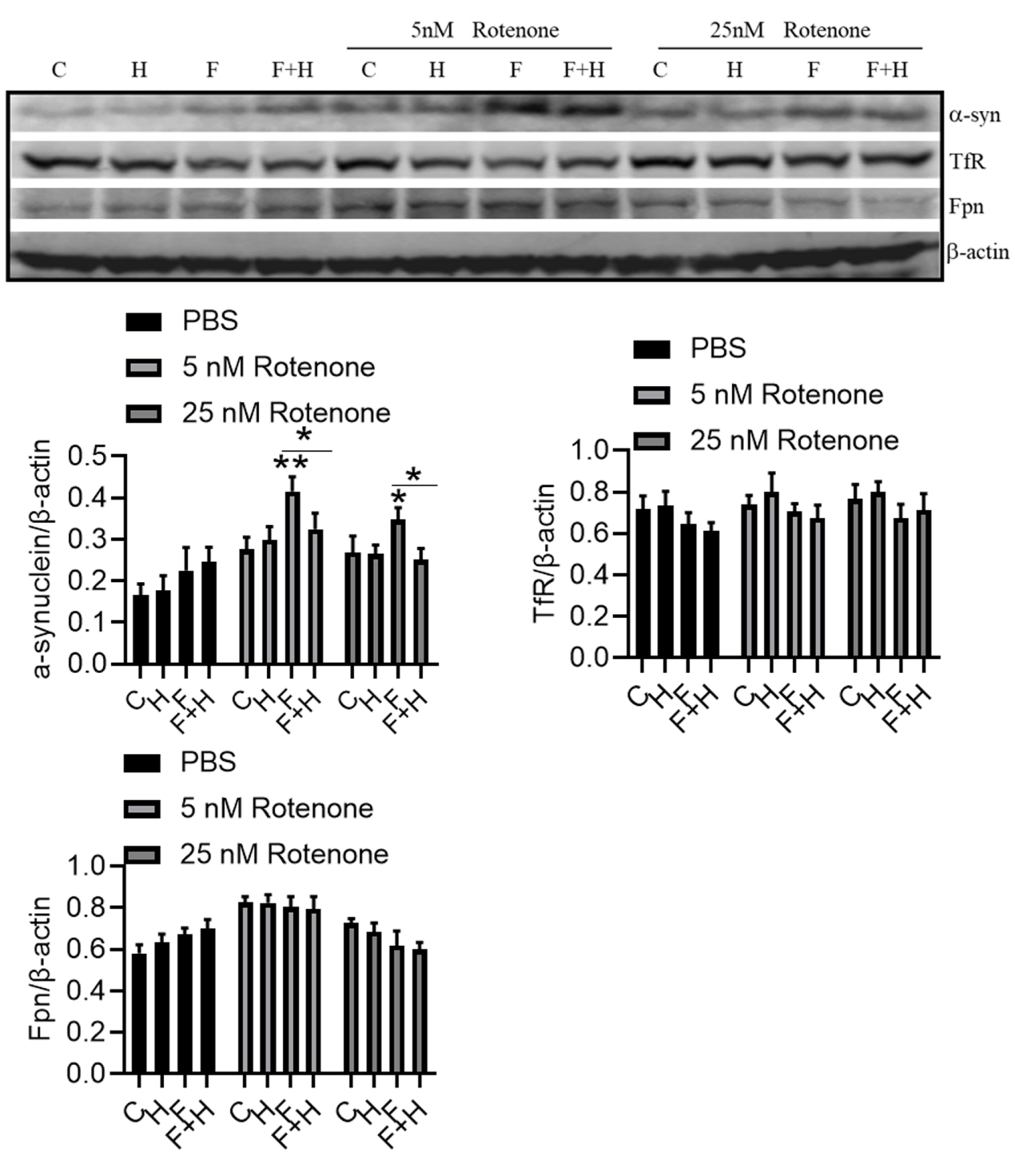


**Fig. S2** α-syn expression was increased in both Ferric Ammonium Ciltete (FAC) or rotenone treatment alone and treatment together. C: control; H: hepcidin; F: FAC, the concentration of FAC and hepcidin was the same with our study in the manuscript. The expression of α-syn was increased significantly both treated with FAC and 5 or 25 nM rotenone when compared to control. There was no changes of α-syn expression after treating with hepcidin alone both under physiological and 5 or 25 nM rotenone treatment. The expression of Fpn was not changed significantly among different groups. * p <0.05, ** p<0.05 compared to the control; * p<0.05 compared to the indicated group.

**Fig. S3** The iron content was increased significantly after treating with FAC when compared with control in SH-SY5Y cells.

**Fig. S4** Cell viability after treating with 100 nM hepcidin for 3 days by MTT method in SH-SY5Y cells (n=4 independent experiments).
