# Supplementary figures and images for: Hepcidin Decreases Rotenone-Induced α-Synuclein Accumulation via Autophagy in SH-SY5Y Cells
Source: Front Mol Neurosci. 2020 Oct 16;13:560891. doi: 10.3389/fnmol.2020.560891 (PMC7596286; doi:10.3389/fnmol.2020.560891)

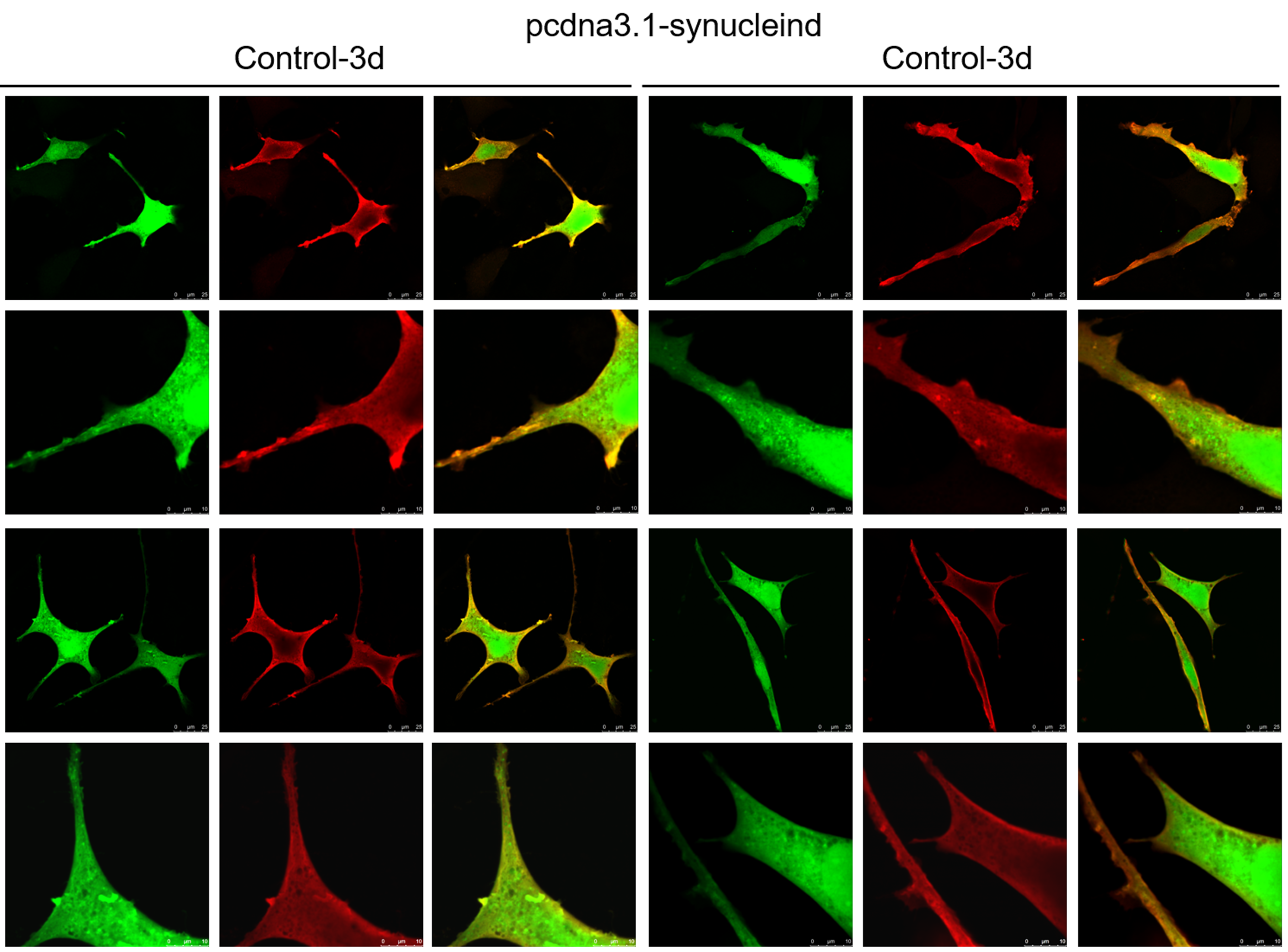

Supplement: Supplementary file 2 [file Data_Sheet_2.ZIP › Supplementary data/Control-alpha-synuclein.tif]

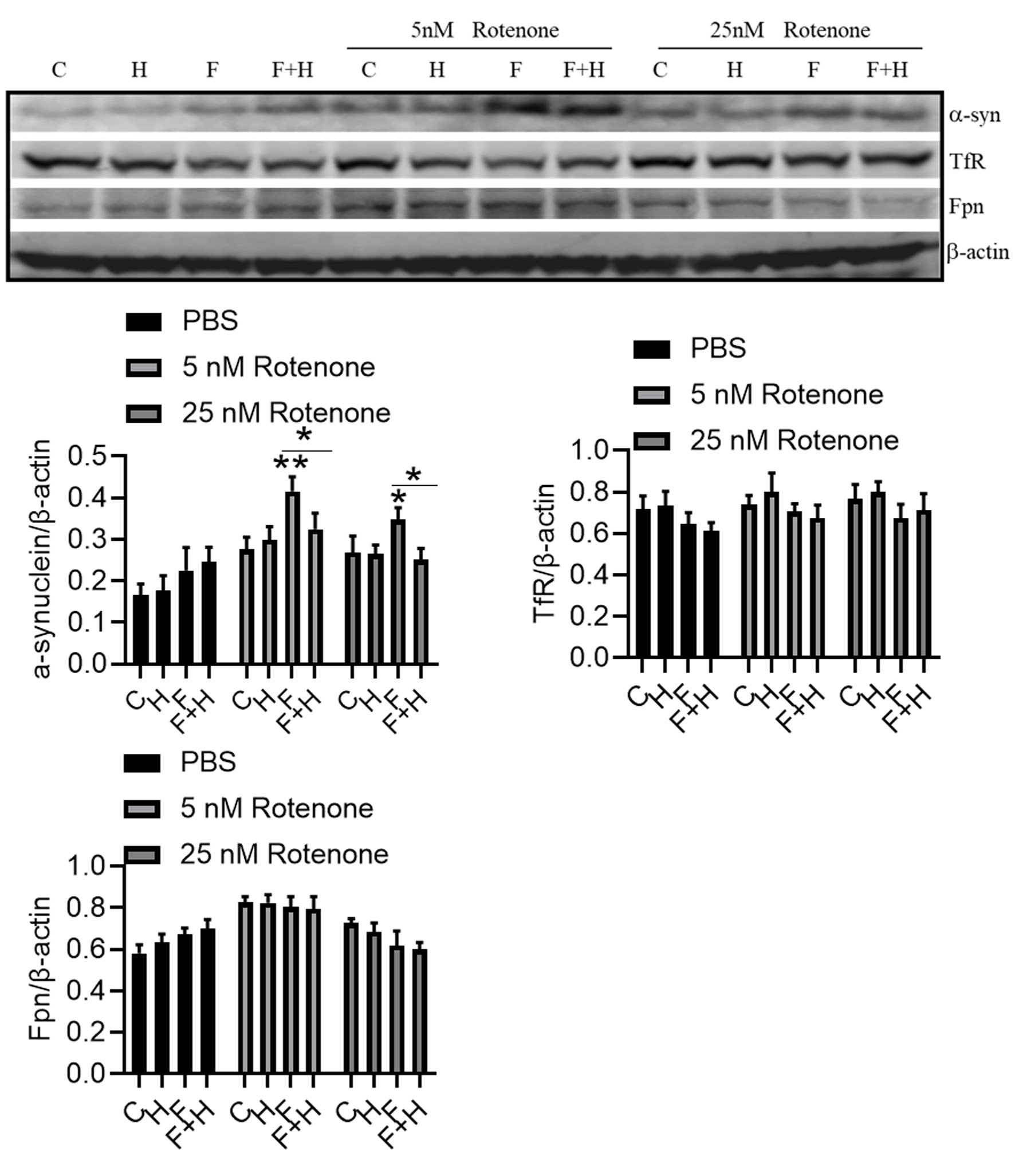

Supplement: Supplementary file 2 [file Data_Sheet_2.ZIP › Supplementary data/Fig S2.tif]

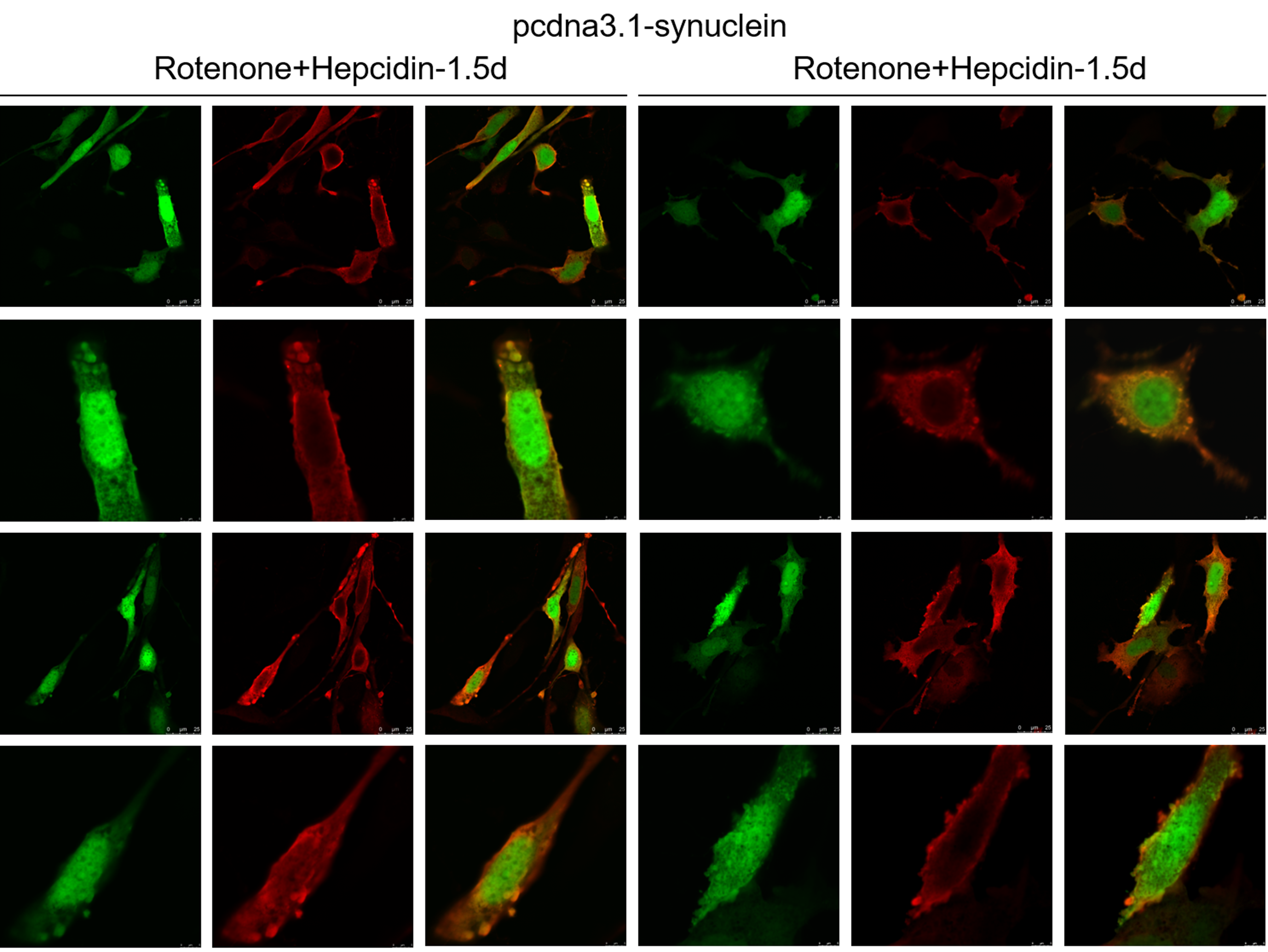

Supplement: Supplementary file 2 [file Data_Sheet_2.ZIP › Supplementary data/Rotenone+Hepcidin-1.5d.tif]

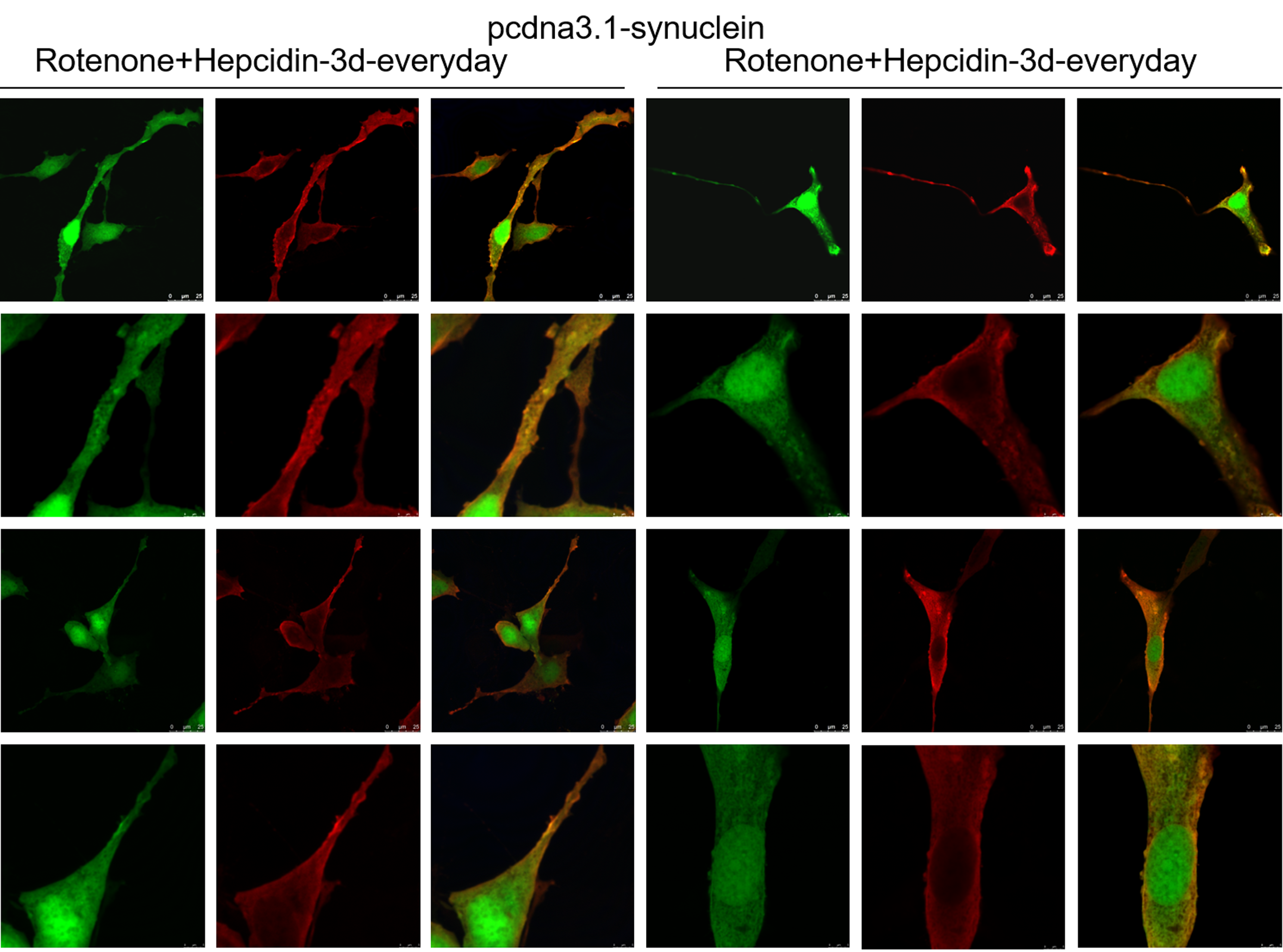

Supplement: Supplementary file 2 [file Data_Sheet_2.ZIP › Supplementary data/Rotenone+Hepcidin-3d.tif]

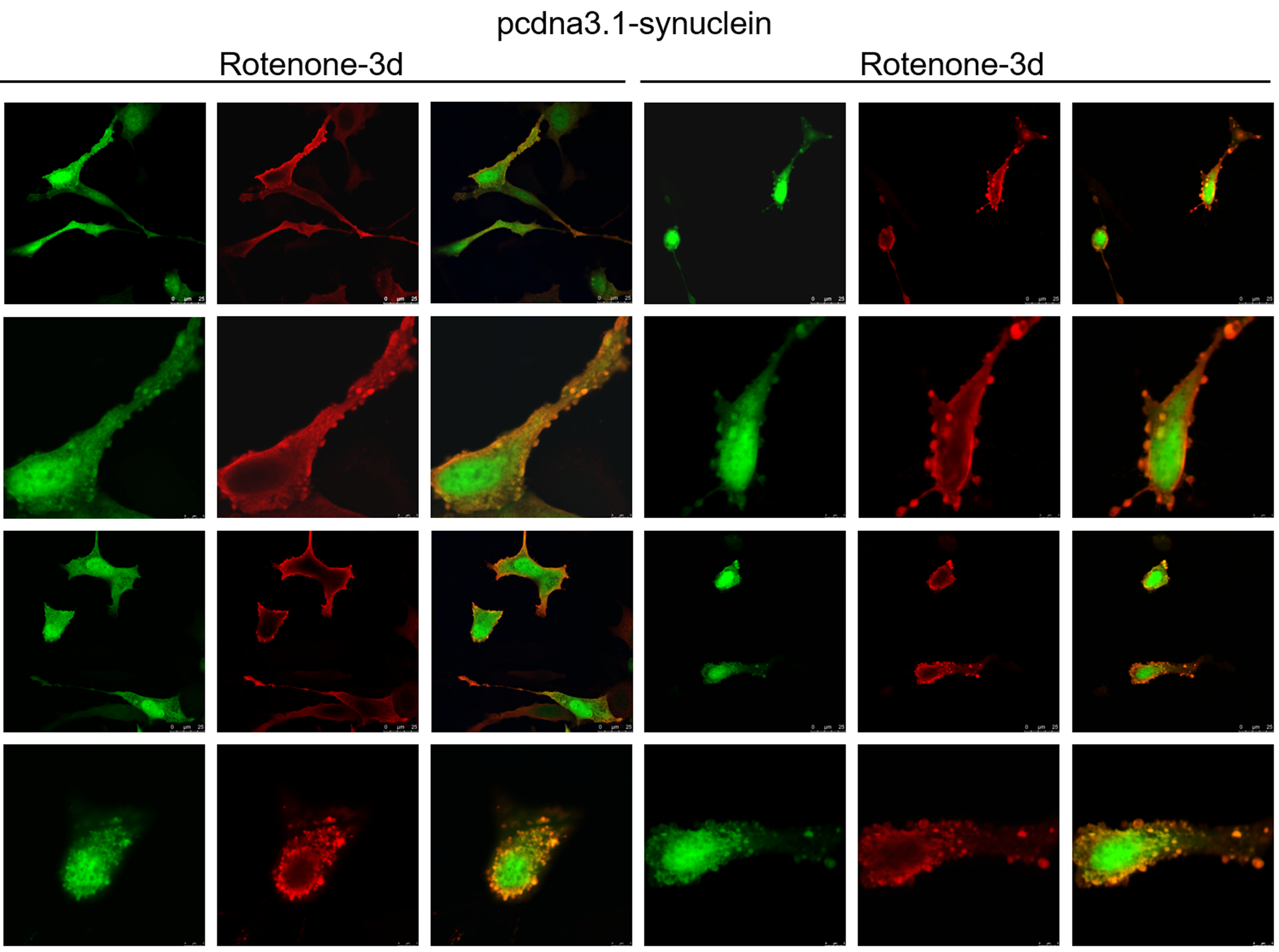

Supplement: Supplementary file 2 [file Data_Sheet_2.ZIP › Supplementary data/Rotenone-3d-alpha-synuclein.tif]
